# Effects of Model-Based Sexual Counseling on Sexual Function and Sexual Life in Women with Breast Cancer: A Systematic Review and Meta-Analysis

**DOI:** 10.3390/healthcare14203516

**Published:** 2026-10-19

**Authors:** Orçun Yalav, Derya Gezer, Seçil Taylan

**Affiliations:** 1 Department of General Surgery, Faculty of Medicine, Cukurova University, Adana 01330, Türkiye; oyalav@cu.edu.tr; 2 Department of Nursing, Faculty of Health Sciences, Tarsus University, Mersin 33400, Türkiye; 3 Department of Surgical Diseases Nursing, Kumluca Faculty of Health Sciences, Akdeniz University, Antalya 07350, Türkiye; seciltaylan@akdeniz.edu.tr

**Keywords:** breast cancer, sexual counseling, sexual health, sexual function, sexuality, nursing

## Abstract

**Highlights:**

**What are the main findings?**

- Model-based sexual counseling was associated with a statistically significant pooled improvement in overall sexual life in women with breast cancer; however, substantial between-study heterogeneity and very low certainty of evidence limit confidence in this finding.
- A statistically significant pooled improvement was also observed for sexual function, with the largest pooled effects at 6–8 weeks and significant effects at 12 weeks; however, the certainty of evidence was very low.
- PLISSIT-specific subgroup effects were not statistically significant; this finding should not be interpreted as evidence that the PLISSIT model is ineffective.

**What are the implications of the main findings?**

- The findings suggest that theoretically structured sexual counseling may have potential as a component of comprehensive breast cancer care; however, the current evidence is insufficient to establish a reliable clinical benefit or support definitive recommendations for practice.
- Standardized counseling protocols and larger, geographically diverse randomized controlled trials with longer follow-up are needed to determine the comparative and sustained effects of different counseling models.

**Abstract:**

**Background/Objectives:** Sexual dysfunction is a common and persistent concern among women with breast cancer. Although various interventions have been developed to address sexual health, evidence regarding counseling interventions structured around theoretical models remains limited. This systematic review and meta-analysis aimed to evaluate the effects of model-based sexual counseling on overall sexual life and sexual function in women with breast cancer. **Methods:** The review was conducted in accordance with the Cochrane Handbook and PRISMA guidelines and was prospectively registered in PROSPERO (CRD420251004532). Five electronic databases were searched for controlled intervention studies published up to February 2025. Risk of bias was assessed using the Cochrane Risk of Bias tool (RoB 1) for randomized controlled trials and ROBINS-I for nonrandomized studies. Random-effects meta-analyses were performed using Comprehensive Meta-Analysis version 3, with effect sizes expressed as Hedges’ *g* and 95% confidence intervals (CIs). **Results:** Ten controlled intervention studies involving 779 participants were included, comprising seven randomized controlled trials and three nonrandomized controlled intervention studies. Model-based counseling was associated with statistically significant pooled effects on overall sexual life (*g* = 1.86, 95% CI 0.94–2.77; *p* < 0.001; *I*^2^ = 95.93%) and sexual function (*g* = 2.11, 95% CI 0.94–3.28; *p* < 0.001; *I*^2^ = 97.08%). PLISSIT-based counseling showed no statistically significant pooled effects. Time-based analyses showed the largest effects at 6–8 weeks, with statistically significant effects persisting at 12 weeks. Substantial heterogeneity was observed across analyses. **Conclusions:** Model-based sexual counseling may have beneficial effects on overall sexual life and sexual function in women with breast cancer; however, the very low certainty of evidence and substantial heterogeneity limit confidence in these findings. Further well-designed randomized controlled trials using standardized interventions and longer follow-up are needed.

## 1. Introduction

Breast cancer is a malignant disease characterized by the uncontrolled growth of abnormal cells in breast tissue and represents a major global health burden among women. According to GLOBOCAN 2022 estimates, female breast cancer accounted for 11.6% of all newly diagnosed cancers worldwide, making it the second most commonly diagnosed cancer globally [1]. Its incidence has increased across many populations [2]. Advances in diagnosis and systemic treatment have substantially improved outcomes, with the 5-year survival rate reaching approximately 90% [3]. As survival improves, increasing attention has shifted toward the long-term consequences of cancer and its treatment, including physical and functional effects, as well as broader survivorship and quality-of-life concerns [4].

Sexual dysfunction is a common and persistent concern among women with breast cancer and may develop during treatment and persist into survivorship [5,6,7,8]. Sexual function is a multidimensional component of sexual health that encompasses sexual desire, arousal, lubrication, orgasm, satisfaction, and pain [9]. In a meta-analysis of 19 studies involving 2684 women with breast cancer, Jing et al. [10] reported a pooled prevalence of female sexual dysfunction of 73.4%, based on the Female Sexual Function Index (FSFI), which assesses desire, arousal, lubrication, orgasm, satisfaction, and pain. However, the sexual experiences of women with breast cancer extend beyond sexual function and may also encompass sexual satisfaction, intimacy, emotional well-being, body image, and relationship-related aspects [5,6,7,8]. In the present review, these interconnected dimensions are considered within the broader context of sexual life. Both sexual function and broader aspects of sexual life may be adversely affected by surgery, chemotherapy, radiotherapy, endocrine therapy, treatment-induced menopause, body image changes, vaginal atrophy, and vaginal dryness [11].

Despite its importance to women with breast cancer [12], sexual health remains insufficiently addressed in routine cancer care [13,14]. Clinical guidelines recommend psychosocial and/or psychosexual interventions to address sexual problems among people with cancer [15]. Accordingly, sexual counseling and education are important components of comprehensive survivorship care and provide a basis for structured interventions aimed at addressing the multidimensional sexual health needs of women with breast cancer.

Model-based sexual counseling provides a systematic framework for identifying sexual concerns and tailoring interventions to individual needs [5,14]. Models such as PLISSIT and BETTER structure the communication and counseling process, whereas theory-based approaches such as self-efficacy and self-regulation target cognitive and behavioral processes that may influence sexual adjustment. Such structured approaches may facilitate the systematic delivery of sexual counseling. However, the extent to which different model-based approaches produce consistent effects on sexual health outcomes remains unclear.

Several approaches have been investigated to improve sexual health in women with breast cancer. Previous systematic reviews and meta-analyses have evaluated PLISSIT-based sexual counseling [16], psychological interventions [17,18], and nursing interventions [18,19,20]. However, these reviews have largely focused on specific intervention types or individual counseling models, leaving uncertainty regarding the overall effectiveness of sexual counseling interventions structured around different theoretical models. Moreover, counseling models differ in their theoretical foundations and intervention strategies, and their effects may vary across follow-up periods. Examining these differences is important for determining whether intervention effects are consistent across counseling approaches and over time. Therefore, this systematic review and meta-analysis aimed to evaluate the effects of model-based sexual counseling on overall sexual life and sexual function in women with breast cancer and, where sufficient data were available, to explore whether these effects varied according to counseling model and follow-up period.

Research Questions

1. What is the effect of model-based sexual counseling on overall sexual life among women with breast cancer?
2. What is the effect of model-based sexual counseling on sexual function among women with breast cancer?

## 2. Methods

### 2.1. Design

This systematic review and meta-analysis was conducted in accordance with the Cochrane Handbook for Systematic Reviews of Interventions and reported in accordance with the Preferred Reporting Items for Systematic Reviews and Meta-Analyses (PRISMA) 2020 statement [21]. The review protocol was prospectively registered with the International Prospective Register of Systematic Reviews (PROSPERO; CRD420251004532). The PICOS framework (Population, Intervention, Comparator, Outcomes, and Study Design) was used to formulate the research question and define the eligibility criteria [22] (Table 1).

### 2.2. Eligibility Criteria

Studies were eligible if they (1) included women diagnosed with breast cancer; (2) evaluated a model- or theory-based sexual counseling intervention; (3) assessed overall sexual life and/or sexual function using sexual health-specific measures; (4) included a control or comparison group receiving standard care, usual care, placebo, or no intervention; and (5) employed a controlled quantitative intervention design, including randomized controlled trials and quasi-experimental controlled studies. Partner participation in the counseling intervention was permitted, provided that sexual health outcomes for women with breast cancer were reported separately. Studies published in languages other than English, those without a control or comparison group, those without sexual health-specific outcome measures, or those without separately extractable outcomes for women with breast cancer were excluded.

For the purposes of this meta-analysis, “overall sexual life” was defined as a broad outcome category encompassing related but conceptually distinct sexual health constructs, including sexual satisfaction, sexual behavior, sexual quality of life, and sexual function, as assessed using validated sexual-health-specific measures. These constructs were not considered psychometrically equivalent. Accordingly, the pooled estimate for overall sexual life was interpreted as an exploratory synthesis of related sexual health outcomes rather than as an estimate of a single homogeneous construct. Within each study, only one effect estimate was included in the overall analysis to avoid double-counting. When more than one eligible measure was reported, the measures were classified according to their underlying construct and combined only when they represented the same outcome domain. Sexual function was additionally analyzed as a separate outcome using sexual-function-specific measures.

### 2.3. Search Strategy

**Figure 1:**
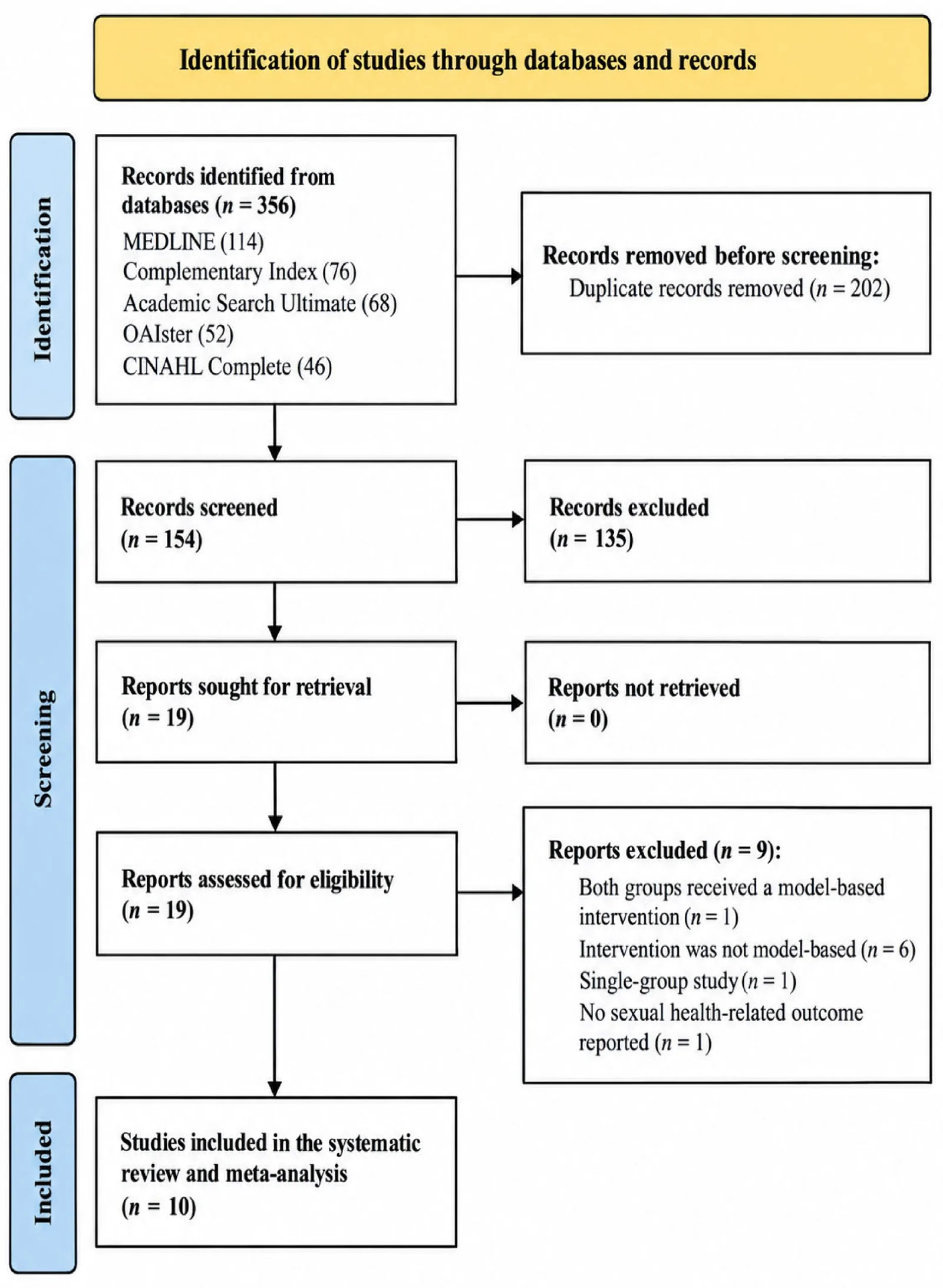
PRISMA flow diagram.

Five electronic databases (MEDLINE, Complementary Index, Academic Search Ultimate, OAIster, and CINAHL) were systematically searched on 10 March 2025 for eligible studies published through February 2025. These databases were selected to provide coverage of biomedical, nursing and allied health, multidisciplinary, and grey literature sources relevant to breast cancer and sexual health. The same search strategy was applied across all five databases. The search combined terms related to breast cancer, sexual health, counseling and theoretical models, and study design using Boolean operators, with all search terms restricted to the abstract field (AB). The complete search terms are presented in Table 2. The reference lists of eligible studies were also manually searched to identify additional relevant studies. The study selection process is presented in the PRISMA flow diagram (Figure 1).

### 2.4. Selection of Studies

EndNote X8 (Clarivate Analytics, Philadelphia, PA, USA) was used to manage the retrieved records and remove duplicates. Two reviewers independently screened studies in three stages: title, abstract, and full-text assessment. Studies of uncertain eligibility were retained for the subsequent screening stage. Disagreements regarding eligibility were resolved through discussion and consensus between the reviewers. Studies meeting all eligibility criteria after full-text assessment were included in the systematic review and meta-analysis.

### 2.5. Data Extraction

Data were extracted using a pilot-tested standardized form. The extracted data included author, publication year, country, study duration, sample size, intervention characteristics, intervention frequency, assessment intervals, and post-intervention outcome measurements.

### 2.6. Risk of Bias Assessment

Two reviewers independently assessed the risk of bias according to study design. The seven randomized controlled trials were evaluated using the Cochrane Risk of Bias tool (RoB 1) [23]. Seven domains were assessed: random sequence generation, allocation concealment, blinding of participants and personnel, blinding of outcome assessment, incomplete outcome data, selective reporting, and other potential sources of bias. Each domain was judged as having a low, unclear, or high risk of bias. The three nonrandomized controlled intervention studies were assessed separately using the Risk Of Bias In Non-randomized Studies of Interventions (ROBINS-I) tool. The assessed domains included bias due to confounding, selection of participants, classification of interventions, deviations from intended interventions, missing data, measurement of outcomes, and selection of the reported result. ROBINS-I judgments were classified as having low, moderate, serious, or critical risk of bias, or as having no information, when appropriate. Disagreements between the reviewers were resolved through discussion and consensus.

### 2.7. Certainty of Evidence

The certainty of evidence for the principal outcomes was assessed using the GRADE approach, considering risk of bias, inconsistency, indirectness, imprecision, and publication bias. Certainty was classified as high, moderate, low, or very low.

### 2.8. Statistical Analysis

Two outcomes were synthesized: overall sexual life and sexual function. Overall sexual life was defined a priori as a broad outcome category encompassing related but not psychometrically identical dimensions of sexual health assessed using validated sexual health-specific measures. Because these outcomes were assessed using different instruments and scales across studies, standardized mean differences were used to synthesize them on a common metric. However, statistical standardization does not eliminate conceptual differences among the underlying constructs. Therefore, the pooled estimate for overall sexual life was interpreted as a broad synthesis of related sexual health outcomes rather than as an estimate of a single homogeneous construct. Sexual function was additionally analyzed as a separate outcome using sexual function-specific measures.

Meta-analyses were performed using Comprehensive Meta-Analysis (CMA) version 3 (Biostat, Inc., Englewood, NJ, USA) [24]. Hedges’ g with 95% confidence intervals (CIs) was calculated from post-intervention scores using random-effects models [25]. When a study reported multiple eligible measures within the same outcome domain, these measures were combined to obtain a single study-level effect estimate, ensuring that each study contributed only one effect estimate to the overall meta-analysis. When multiple follow-up assessments were available, effect estimates were analyzed separately according to the predefined follow-up categories (post-intervention to 4 weeks, 6–8 weeks, and 12 weeks), with only one estimate per study included in each time-specific analysis. For multi-arm trials, only one eligible intervention-versus-control comparison was included in each pooled analysis. Thus, shared control groups were not included more than once, avoiding double-counting of participants and artificial inflation of statistical precision.

Heterogeneity was assessed using Cochran’s Q, I^2^, and τ^2^ statistics [26]. To assess the robustness of the pooled estimates and determine whether the observed heterogeneity was disproportionately influenced by any individual study, leave-one-out sensitivity analyses were performed by sequentially excluding one study at a time and recalculating the pooled effect estimate and heterogeneity statistics. Subgroup analyses were conducted for PLISSIT-based interventions and by follow-up period (post-intervention to 4 weeks, 6–8 weeks, and 12 weeks). Publication bias was assessed using Kendall’s tau and Egger’s regression test [27]. Statistical significance was set at *p* < 0.05.

## 3. Results

### 3.1. Search Results and Study Selection

The systematic search of five electronic databases identified 356 records. After duplicate removal, 154 unique records underwent title and abstract screening, of which 19 were assessed in full text. Following assessment according to the eligibility criteria, 10 controlled intervention studies, comprising seven randomized controlled trials and three nonrandomized controlled intervention studies, were included in the systematic review and meta-analysis. The study selection process is presented in the PRISMA flow diagram (Figure 1).

### 3.2. Risk of Bias Assessment

The risk-of-bias assessment was conducted separately according to study design. Among the randomized controlled trials assessed using the Cochrane RoB 1 tool, most domains were judged to be at low risk of bias. Unclear risk was observed mainly for blinding of participants and personnel [28,29], blinding of outcome assessment [30], and, in some studies, other potential sources of bias [28,31,32]. No study was judged to be at high risk of bias in any of the assessed domains.

**Figure 2:**
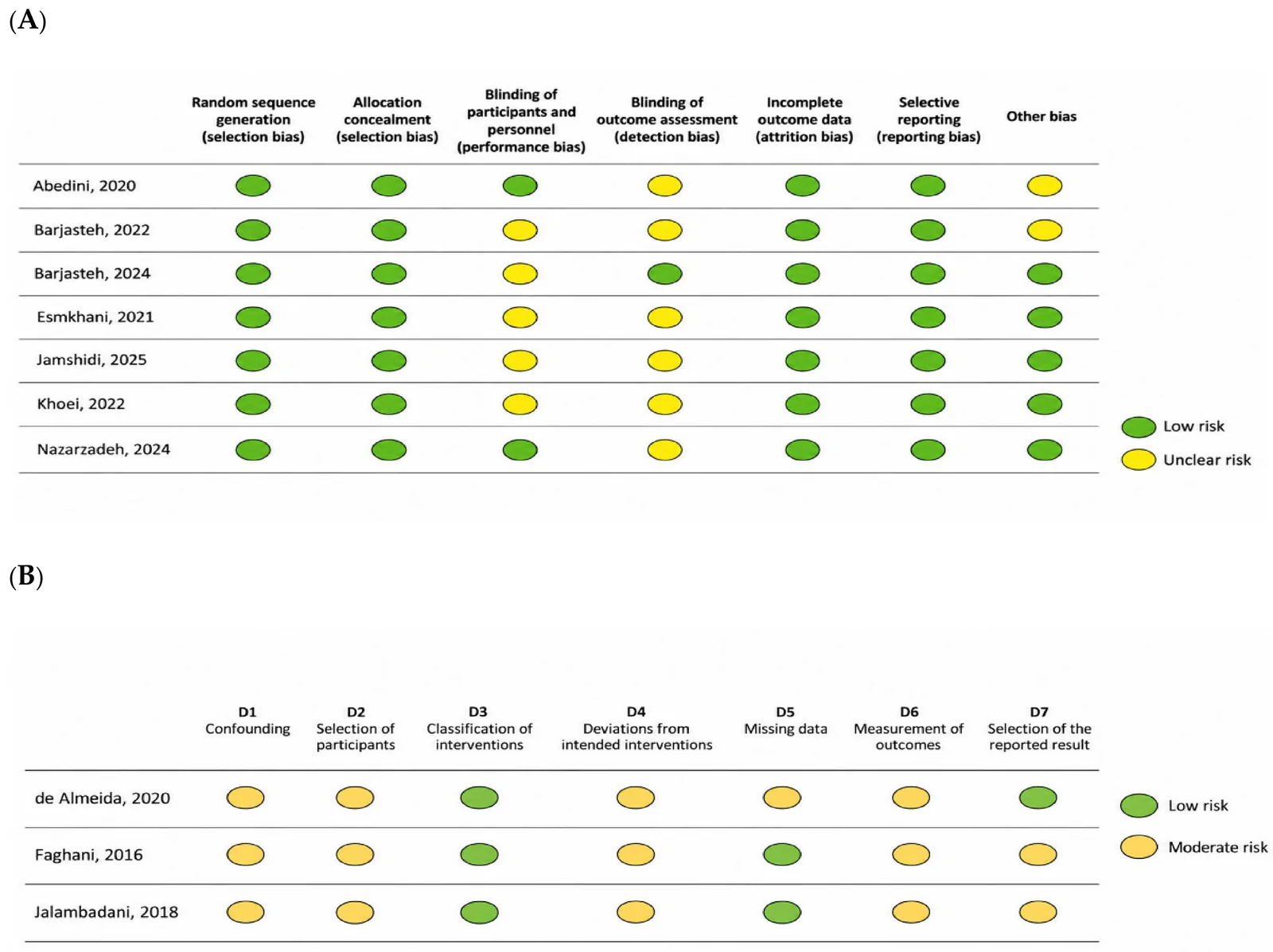
Risk-of-bias assessment of the included studies. (**A**) Risk-of-bias assessment of randomized controlled trials using the Cochrane Risk of Bias tool (RoB 1) [28,29,30,31,34,35,36]. (**B**) Risk-of-bias assessment of non-randomized studies using ROBINS-I [32,33,37].

The three nonrandomized studies were evaluated using ROBINS-I. These studies were generally judged to have a moderate risk of bias in the domains of confounding, participant selection, deviations from intended interventions, and outcome measurement. The risk of bias related to the classification of interventions was low across all three studies. For missing data, the risk of bias was judged to be low in Faghani et al. (2016) [32] and Jalambadani et al. (2018) [33] and moderate in de Almeida et al. (2020) [34]. Overall, the nonrandomized studies showed some methodological limitations, but no domain was judged to be at serious or critical risk of bias. The risk-of-bias assessments for the randomized and nonrandomized studies are presented in Figure 2.

### 3.3. Characteristics of the Included Studies

The 10 included controlled intervention studies were published between 2016 and 2025 (Table 3). Nine studies were conducted in Iran [28,29,30,31,32,33,34,35,37], and one was conducted in Brazil [34]. Overall, 779 participants were included (intervention, *n* = 417; control, *n* = 362), with individual study sample sizes ranging from 18 to 120. Two studies used three-group designs comparing PLISSIT with another sexual health intervention and a control condition [35,36].

The interventions were based on several theoretical frameworks. PLISSIT was the most frequently used model [32,34,35,36], followed by Good Enough Sex (GES)-based counseling [30,31]. Other interventions were based on the BETTER model [29], Self-Regulation Model [28], Self-Efficacy Theory [37], and Theory of Planned Behavior [33]. Follow-up assessments varied across studies, ranging from immediate post-intervention assessment to 12 weeks. Study populations included women with breast cancer, breast cancer survivors, and women undergoing mastectomy.

### 3.4. Intervention Outcomes and Subgroup Analyses

The included studies assessed various sexual health outcomes, including dyadic sexual communication [30], reproductive concerns [31], sexual behavior [35], sexual pleasure [36], sexual quality of life [29,32], sexual satisfaction [28,30], and sexual function [29,30,31,32,33,34,36,37]. These outcomes were synthesized as overall sexual life and sexual function, as defined in the Methods.

Four studies evaluated PLISSIT-based counseling [32,34,35,36], allowing subgroup analyses of its effects on both outcomes. Because outcome assessments were conducted at different follow-up periods, additional subgroup analyses were performed for three predefined follow-up periods: post-intervention to 4 weeks, 6–8 weeks, and 12 weeks.

### 3.5. Effect of Model-Based Counseling on Overall Sexual Life

**Figure 3:**
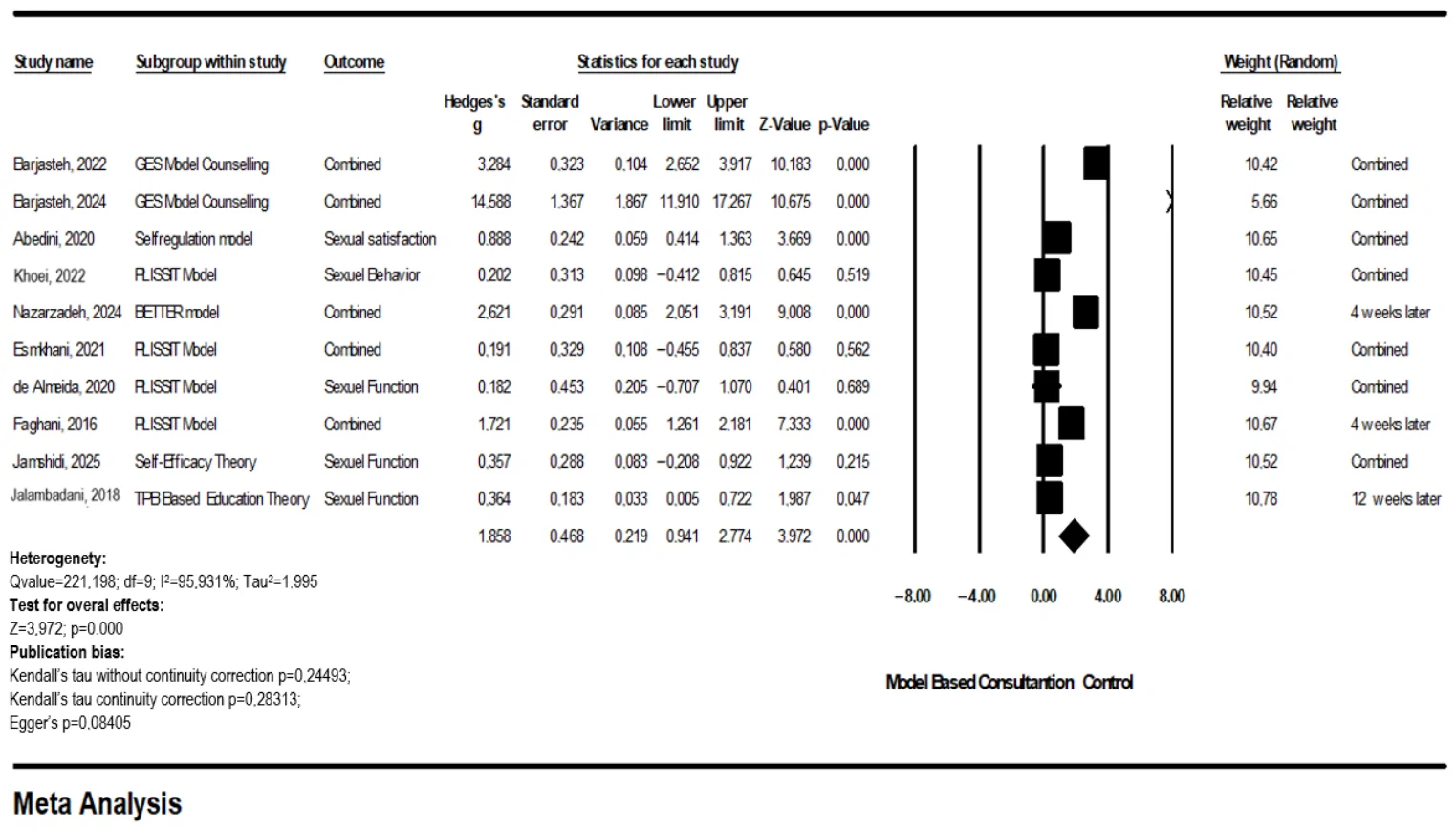
Forest plot of the effect of model-based counseling on overall sexual life. Black squares represent individual study effect-size estimates, with square size proportional to study weight; horizontal lines represent 95% confidence intervals.

Across 10 studies, 729 participants contributed to the overall sexual life meta-analysis (intervention, *n* = 367; control, *n* = 362). Although the total sample across all included studies was 779 participants, 50 participants from additional active intervention arms in two three-arm trials were not included in this pooled comparison to avoid double-counting the shared control groups. Model-based counseling was associated with a statistically significant pooled effect on overall sexual life compared with control conditions (Hedges’ *g* = 1.86, 95% CI [0.94, 2.77], *p* < 0.001). Substantial heterogeneity was observed, *Q*(9) = 221.20, *p* < 0.001, *I*^2^ = 95.93%, τ^2^ = 1.995. Publication bias tests were not statistically significant (Kendall’s tau, *p* = 0.245; Egger’s regression, *p* = 0.084). However, given the small number of included studies, these tests had limited statistical power; therefore, nonsignificant findings should not be interpreted as evidence of the absence of publication bias (Figure 3).

A sensitivity analysis excluding Barjasteh et al. (2024) [30], which showed an unusually large individual effect size, reduced the pooled effect from Hedges’ *g* = 1.86 (95% CI 0.94–2.77) to *g* = 1.12 (95% CI 0.44–1.80), while the overall effect remained statistically significant (*p* = 0.001). Heterogeneity also decreased from *Q*(9) = 221.20, *I*^2^ = 95.93%, and τ^2^ = 1.995 to *Q*(8) = 114.54, *I*^2^ = 93.02%, and τ^2^ = 0.993. Thus, although Barjasteh et al. contributed substantially to the magnitude of the pooled effect and between-study heterogeneity, its exclusion did not alter the direction or statistical significance of the overall finding.

**Figure 4:**
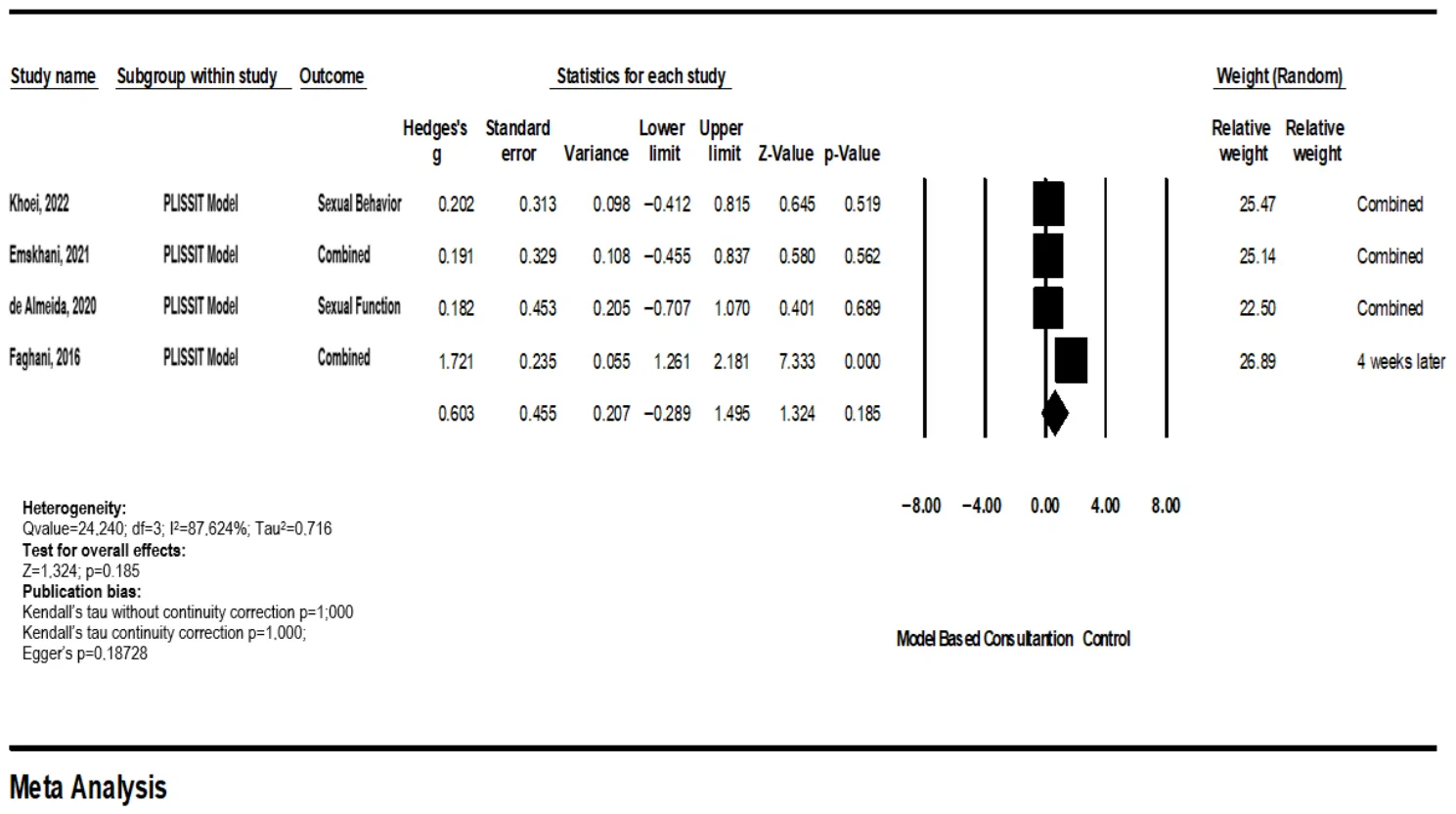
Forest plot of subgroup analysis on the effect of PLISSIT model-based counseling on overall sexual life. Black squares represent individual study effect-size estimates, with square size proportional to study weight; horizontal lines represent 95% confidence intervals.

In the PLISSIT subgroup (four studies; *n* = 196), the pooled effect was not statistically significant (Hedges’ g = 0.60, 95% CI [−0.29, 1.50], *p* = 0.185), and substantial heterogeneity was observed (Q(3) = 24.24, I^2^ = 87.62%, τ^2^ = 0.716) (Figure 4).

**Figure 5:**
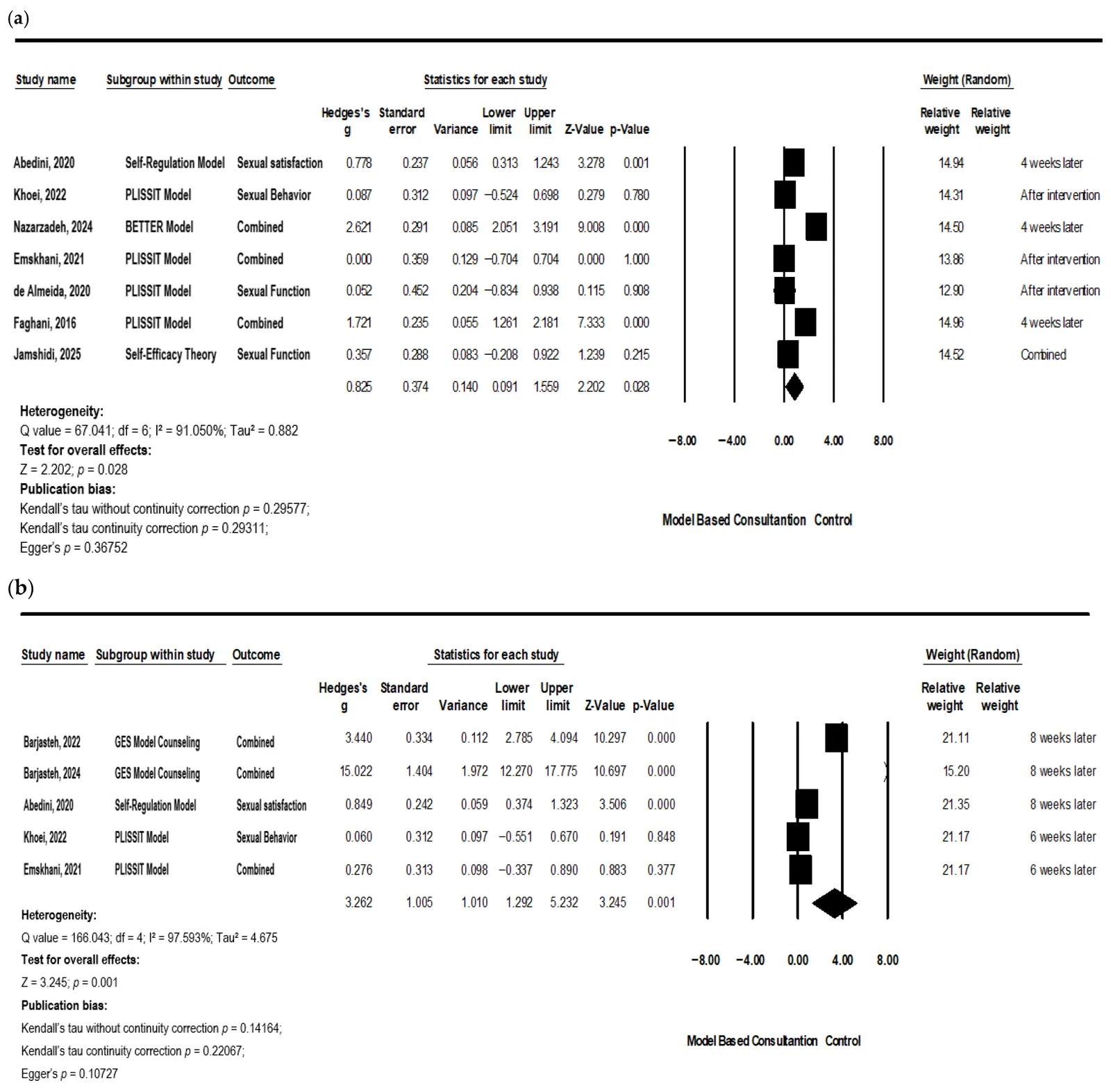

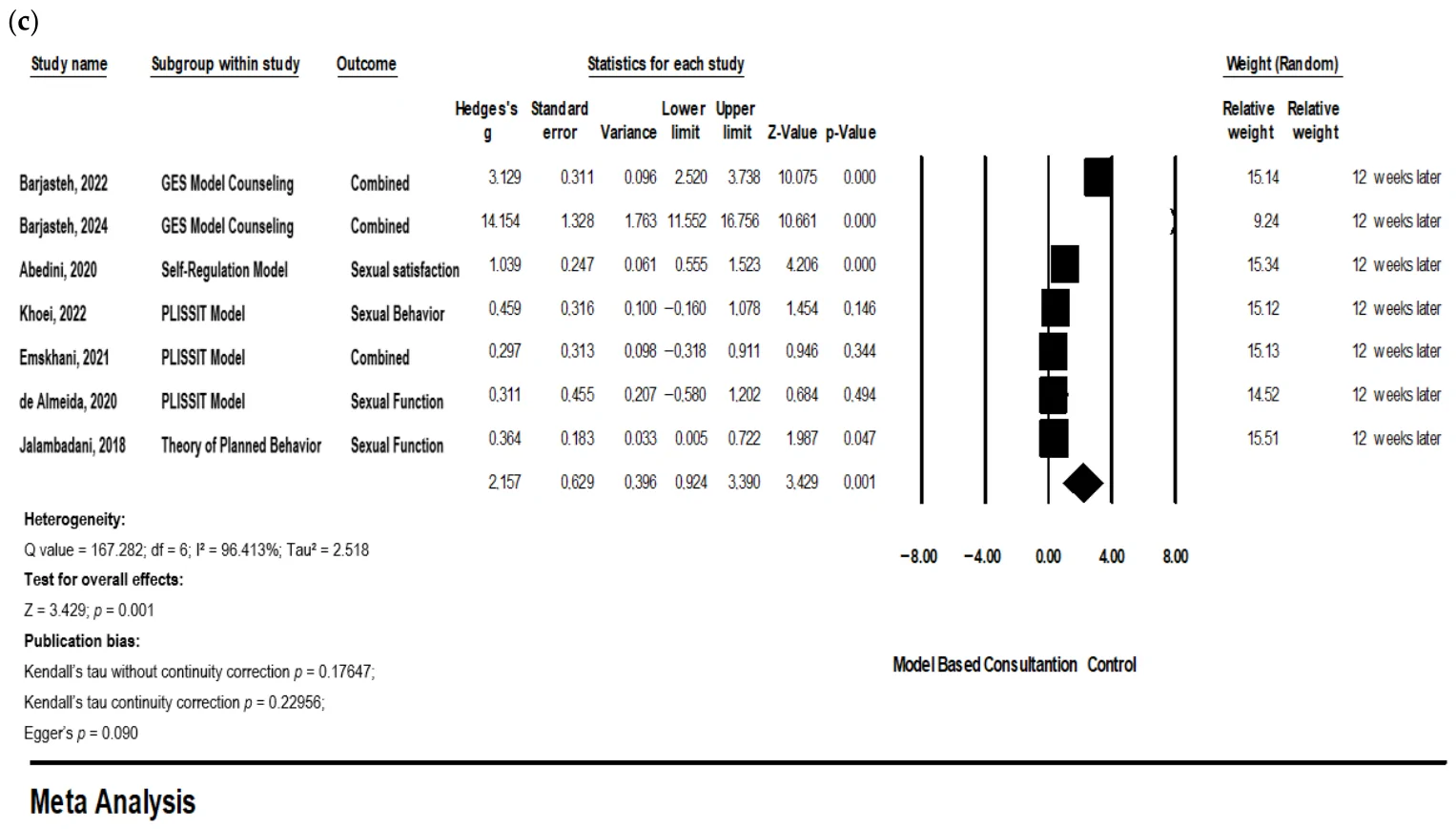
Forest plots of subgroup analysis on the time-dependent effect of model-based counseling on overall sexual life. (**a**)—The effect of model-based counseling on overall sexual life within 4 weeks after the intervention. (**b**)—The effect of model-based counseling on overall sexual life between 4 and 8 weeks after the intervention. (**c**)—The effect of model-based counseling on overall sexual life at the 12th week after the intervention. Black squares represent individual study effect-size estimates, with square size proportional to study weight; horizontal lines represent 95% confidence intervals.

Time-based subgroup analyses included seven studies with 409 participants from post-intervention to 4 weeks, five studies with 363 participants at 6–8 weeks, and seven studies with 501 participants at 12 weeks. Statistically significant pooled effects were observed from post-intervention to 4 weeks (Hedges’ g = 0.83, 95% CI [0.09, 1.56], *p* = 0.028, I^2^ = 91.05%), at 6–8 weeks (g = 3.26, 95% CI [1.29, 5.23], *p* = 0.001, I^2^ = 97.59%), and at 12 weeks (g = 2.16, 95% CI [0.92, 3.39], *p* = 0.001, I^2^ = 96.41%) (Figure 5). Given the differences in study composition across follow-up subgroups and the substantial heterogeneity within each subgroup, these estimates should not be interpreted as evidence of a temporal pattern in intervention effectiveness.

### 3.6. Effect of Model-Based Counseling on Sexual Function

**Figure 6:**
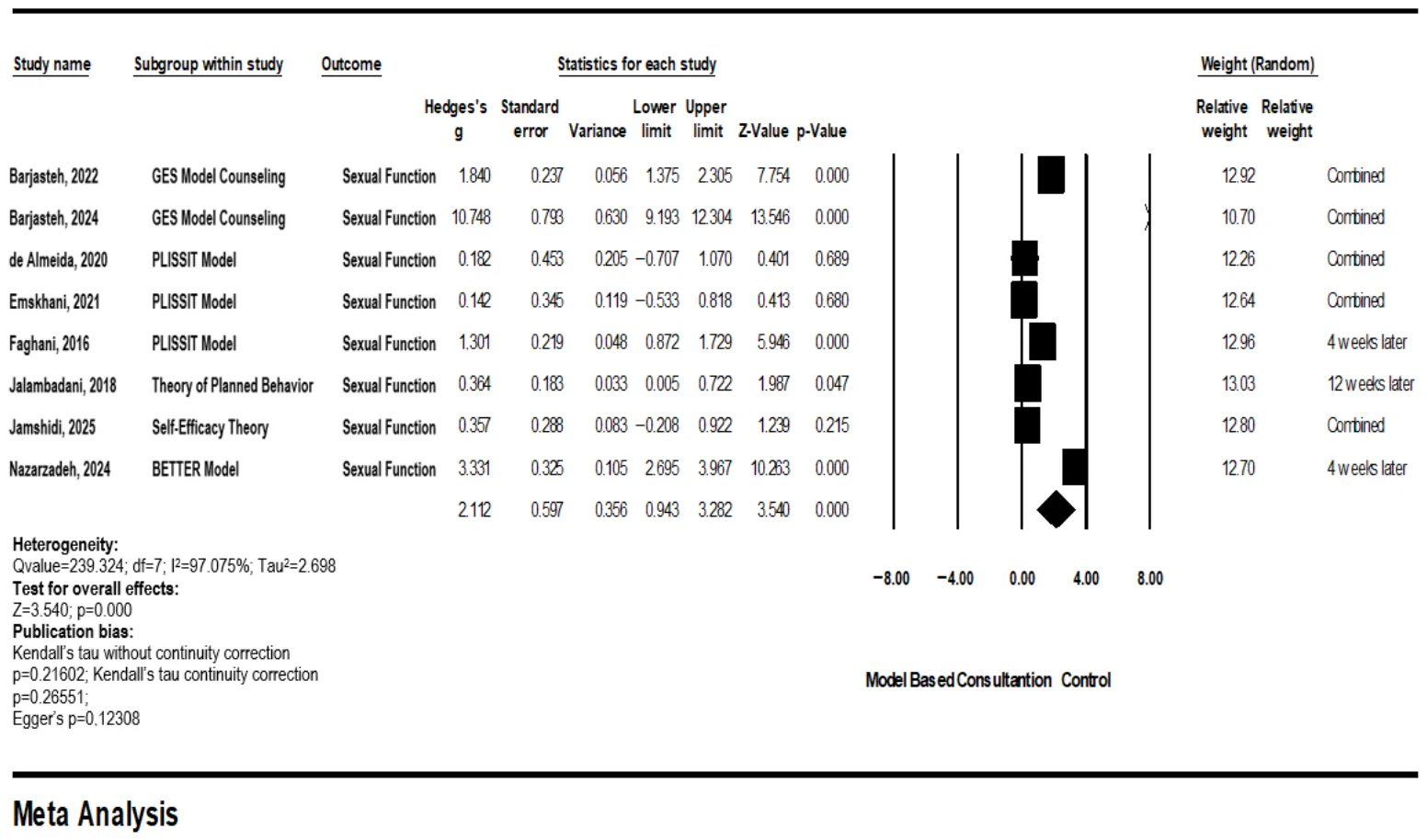
Forest plot of the effect of model-based counseling on sexual function. Black squares represent individual study effect-size estimates, with square size proportional to study weight; hori-zontal lines represent 95% confidence intervals.

Across eight studies involving 616 participants (intervention, *n* = 308; control, *n* = 308), model-based counseling was associated with a statistically significant pooled effect on sexual function compared with control conditions (Hedges’ *g* = 2.11, 95% CI [0.94, 3.28], *p* < 0.001). Substantial heterogeneity was observed, *Q*(7) = 239.32, *I*^2^ = 97.08%, τ^2^ = 2.698. Publication bias tests were not statistically significant (Kendall’s tau, *p* = 0.216; Egger’s regression, *p* = 0.123) (Figure 6). However, given the small number of studies, these tests had limited statistical power; therefore, nonsignificant findings should be interpreted cautiously. Leave-one-out sensitivity analysis showed that substantial heterogeneity persisted after the sequential exclusion of individual studies, indicating that no single study accounted for the observed between-study variability.

**Figure 7:**
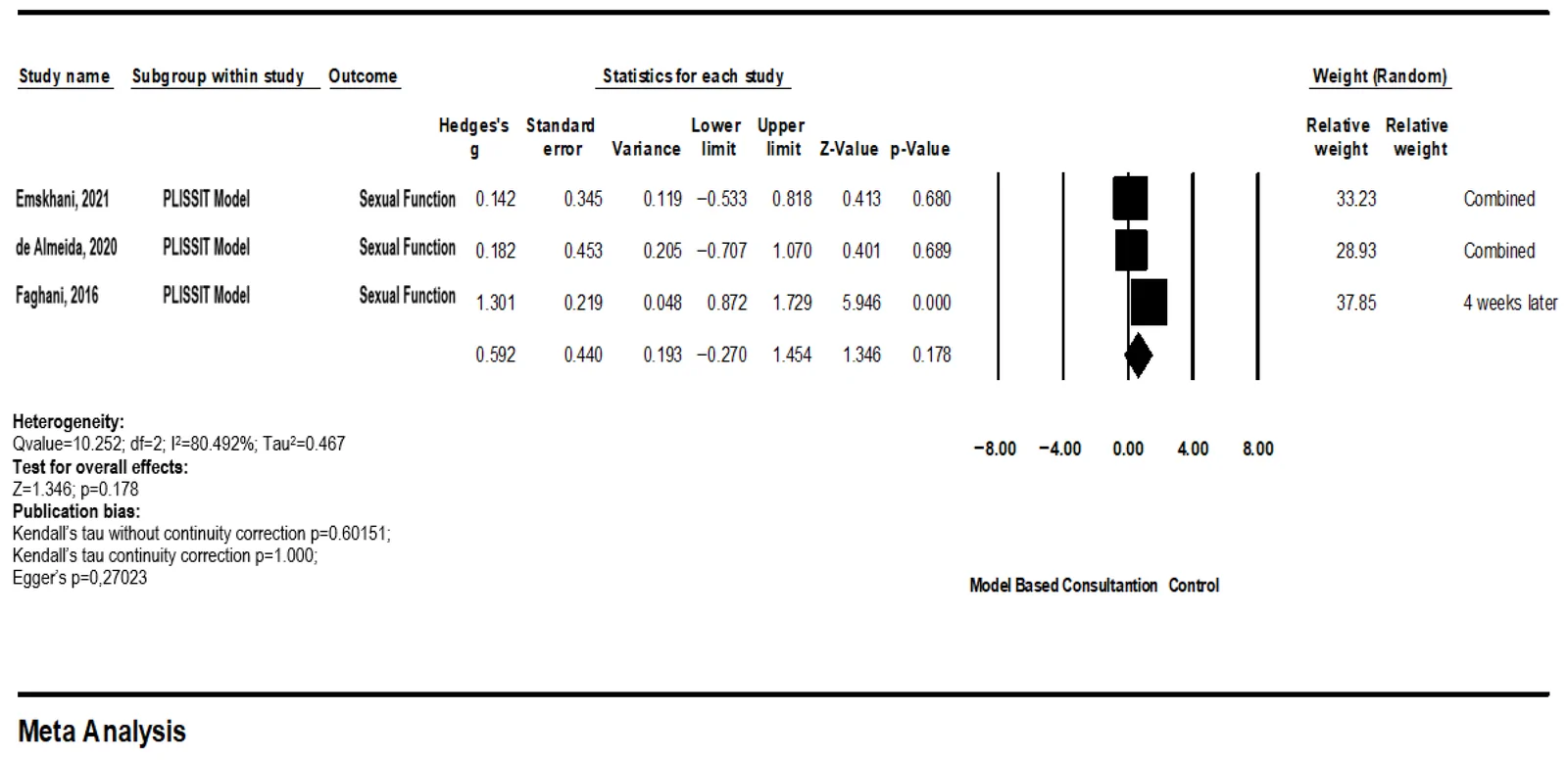
Forest plot of subgroup analysis on the effect of PLISSIT model-based counseling on sexual function. Black squares represent individual study effect-size estimates, with square size proportional to study weight; hori-zontal lines represent 95% confidence intervals.

In the PLISSIT subgroup, the pooled effect was not statistically significant (Hedges’ *g* = 0.59, 95% CI [−0.26, 1.43], *p* = 0.174), with substantial heterogeneity, *Q*(2) = 10.25, *I*^2^ = 80.49%, τ^2^ = 0.467 (Figure 7).

**Figure 8:**
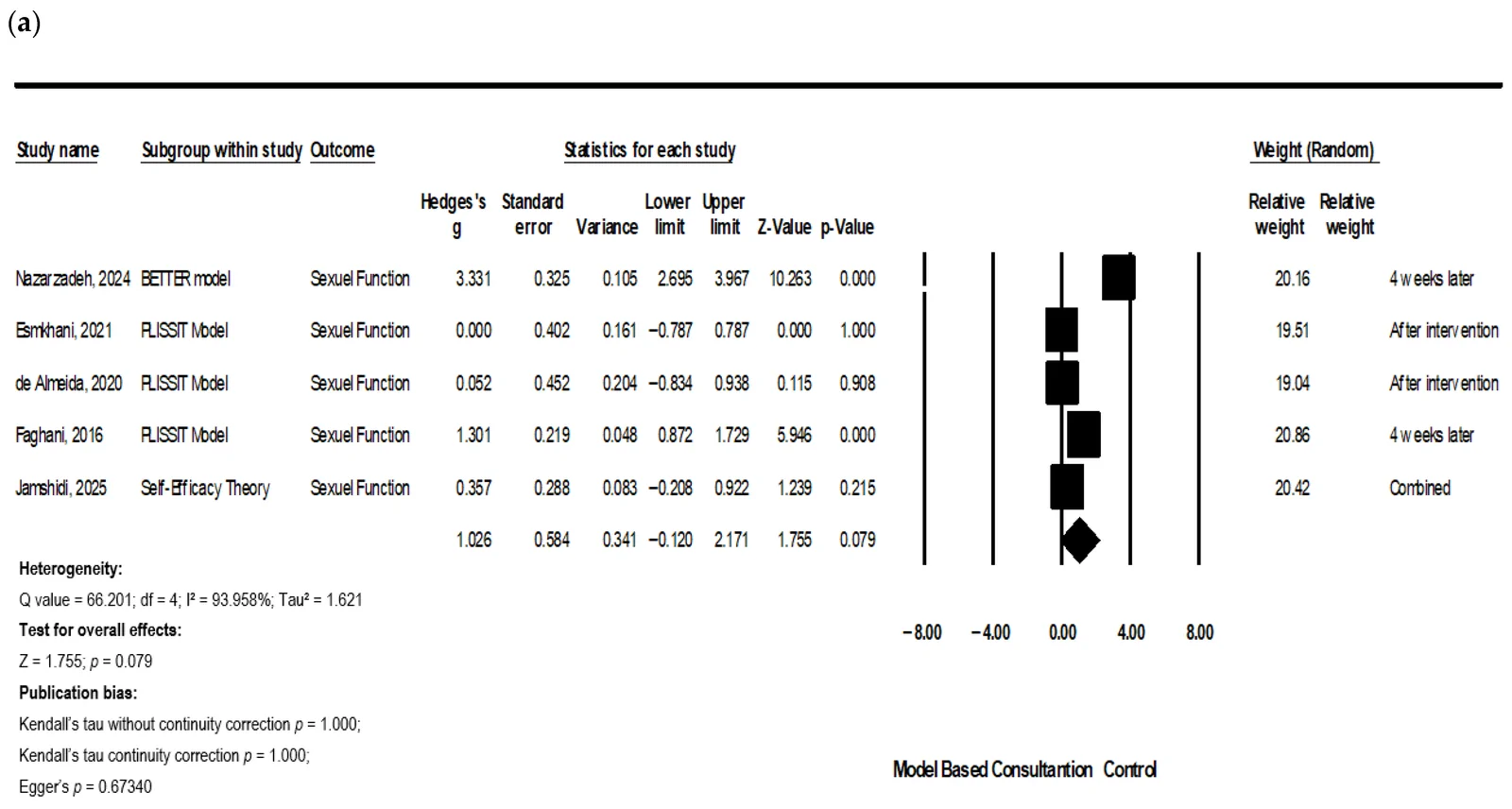

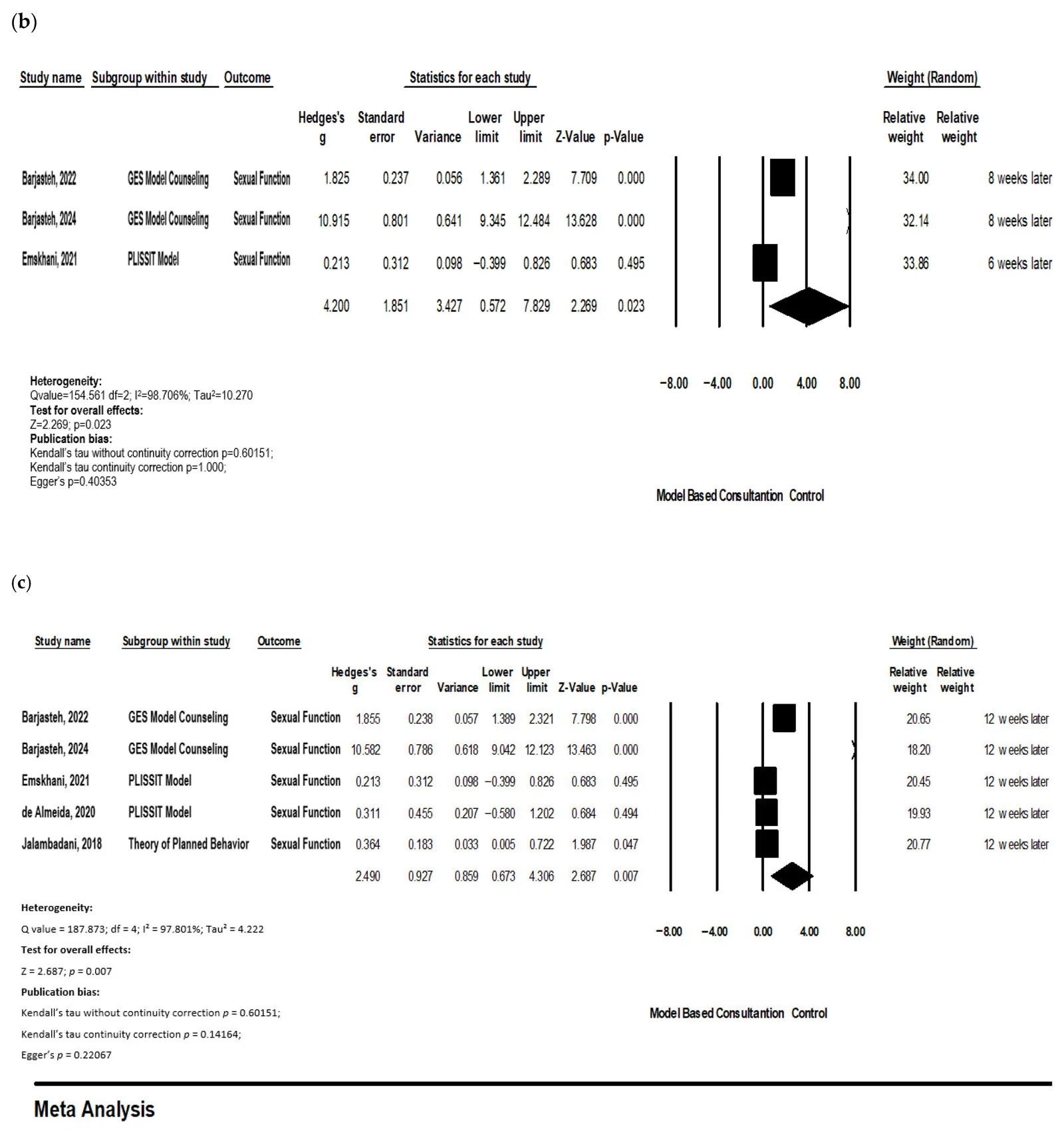
Forest plot of subgroup analysis on the time-dependent effect of model-based counseling on sexual function. (**a**)—The effect of model-based counseling on sexual function within 4 weeks after the intervention. (**b**)—The effect of model-based counseling on sexual function between 4 and 8 weeks after the intervention. (**c**)—The effect of model-based counseling on sexual function at the 12th week after the intervention. Black squares represent individual study effect-size estimates, with square size proportional to study weight; hori-zontal lines represent 95% confidence intervals.

Time-based subgroup analyses included three studies with 228 participants from post-intervention to 4 weeks, three studies with 250 participants at 6–8 weeks, and five studies with 388 participants at 12 weeks. No statistically significant pooled effect was observed from post-intervention to 4 weeks (Hedges’ *g* = 1.03, 95% CI [−0.12, 2.18], *p* = 0.079, *I*^2^ = 93.96%), whereas statistically significant pooled effects were observed at 6–8 weeks (*g* = 4.20, 95% CI [0.56, 7.84], *p* = 0.024, *I*^2^ = 98.71%) and at 12 weeks (*g* = 2.49, 95% CI [0.67, 4.31], *p* = 0.007, *I*^2^ = 97.80%) (Figure 8). The largest pooled effect was observed at 6–8 weeks. Given the small number of studies, publication bias findings for these subgroup analyses were interpreted cautiously.

### 3.7. Certainty of Evidence

The certainty of evidence for the principal outcomes was assessed using the GRADE approach (Table 4). The certainty of evidence was rated as very low for both overall sexual life and sexual function, primarily because of very serious inconsistency and serious indirectness related to the marked geographical concentration of the available evidence.

## 4. Discussion

This systematic review and meta-analysis evaluated the effects of model-based sexual counseling on overall sexual life and sexual function in women with breast cancer. Statistically significant pooled effects were observed for both outcomes; however, substantial between-study heterogeneity was present. In contrast, PLISSIT-specific subgroup analyses did not show statistically significant pooled effects. The inclusion of only 10 controlled intervention studies, comprising seven randomized controlled trials and three nonrandomized controlled intervention studies, also highlights the limited evidence available for model-based sexual counseling in this population.

An important consideration is the variation in how sexual health was conceptualized and measured across the included studies. Although sexual function was assessed in most studies, other dimensions of sexual health were less consistently evaluated. This unequal representation may have contributed to the substantial between-study heterogeneity and should be considered when interpreting the pooled effect estimate for overall sexual life.

Supporting sexual health is an important component of comprehensive care for women with breast cancer. The findings of the present meta-analysis are generally consistent with previous evidence showing beneficial effects of sexual counseling, psychological interventions, and nursing interventions on sexual health outcomes in women with breast cancer [16,17,19,20,38,39]. Individual studies have also reported improvements in sexual function following counseling and education based on Self-Efficacy Theory [37], while psychological, educational, and nursing interventions, including cognitive behavioral therapy, psychoeducation, and psychosexual counseling, have demonstrated beneficial effects on sexual function and related outcomes [17,20]. Together, these findings suggest that structured sexual health interventions may help address the multidimensional sexual concerns experienced by women during and after breast cancer treatment.

The interventions included in this meta-analysis were based on six theoretical frameworks: the PLISSIT model [40], BETTER model [41], GES approach [42], Self-Regulation Model [43], Self-Efficacy Theory [44], and Theory of Planned Behavior [45]. Among these, PLISSIT was the most frequently evaluated model. Although previous studies and reviews have reported beneficial effects of PLISSIT-based interventions on sexual function and related sexual health outcomes [16,38,39], the evidence in women with breast cancer remains limited and heterogeneous. Differences in intervention delivery, duration, outcome measurement, and follow-up periods may have contributed to the variability in observed effects. Further well-designed trials using standardized outcomes and clearly described intervention protocols are therefore needed to clarify the effects of PLISSIT-based counseling in this population.

The pooled effect estimates varied across follow-up subgroups for both overall sexual life and sexual function. However, these differences should not be interpreted as evidence of a temporal pattern in intervention effectiveness because the subgroups included different sets of studies and showed substantial heterogeneity. Previous evidence has also indicated that findings may vary according to the timing of post-intervention assessment [16,20]. Differences in counseling model, intervention intensity, outcome measures, follow-up intervals, and participant characteristics may have contributed to the observed heterogeneity. Further trials using standardized intervention protocols and consistent assessment periods are needed to clarify the effects of model-based sexual counseling over time.

From a clinical perspective, model-based sexual counseling may provide a structured approach to addressing sexual health concerns as part of comprehensive breast cancer care. Nurses and other healthcare professionals may use such approaches to identify sexual health concerns, provide appropriate counseling, and facilitate referral to relevant specialists when needed. However, given the very low certainty of evidence, substantial between-study heterogeneity, and limited evidence for individual counseling models, the pooled findings should not be interpreted as establishing a reliable clinical benefit or supporting the superiority of any specific model. Therefore, integration into clinical practice should be considered cautiously, taking into account individual patient needs, the clinical context, and available resources.

## 5. Strengths and Limitations

This systematic review and meta-analysis has several strengths. The inclusion of controlled intervention studies, systematic risk-of-bias assessment, and evaluation of interventions based on different theoretical models strengthened the methodological rigor of the study. In addition, subgroup analyses according to counseling model and follow-up period provided further insight into potential variations in intervention effects.

Several limitations should also be considered. First, nine of the ten included studies were conducted in Iran, which may limit the geographical and cultural generalizability of the findings. Second, substantial heterogeneity was observed in the overall and subgroup analyses, potentially reflecting differences in counseling models, intervention content and duration, outcome measures, follow-up periods, and participant characteristics. Third, the overall sexual life outcome combined related but conceptually and psychometrically distinct dimensions of sexual health measured using different instruments. Therefore, despite the use of standardized effect sizes, the pooled estimate should be interpreted as a broad synthesis of sexual health-related outcomes rather than as an estimate of a single homogeneous construct. Fourth, the small number of studies, particularly in subgroup analyses, limited the precision of model-specific effect estimates and the ability to assess publication bias reliably. Fifth, the literature search was limited to five electronic databases and did not include other potentially relevant databases such as Embase, Web of Science, Scopus, PsycINFO, or CENTRAL. Restricting the search terms to the abstract field may also have reduced search sensitivity and resulted in potentially eligible studies being missed. Finally, considerable variation in intervention frequency, duration, and delivery indicates a lack of standardized counseling protocols. These limitations warrant cautious interpretation of the pooled effect estimates and highlight the need for larger, geographically diverse controlled studies using standardized intervention protocols and outcome measures.

## 6. Conclusions

This systematic review and meta-analysis suggests that model-based sexual counseling may have beneficial effects on overall sexual life and sexual function in women with breast cancer. However, substantial between-study heterogeneity and the very low certainty of evidence limit confidence in the pooled estimates. PLISSIT-specific subgroup analyses did not demonstrate statistically significant pooled effects; however, these findings should not be interpreted as evidence that the PLISSIT model is ineffective. Overall, the findings suggest a potential role for theoretically structured sexual counseling in breast cancer care, but the current evidence is insufficient to establish a reliable clinical benefit or support the superiority of any specific counseling model. Further well-designed, geographically diverse RCTs using standardized counseling protocols and outcome measures, with longer follow-up periods, are needed to clarify the comparative and sustained effects of different counseling models.

## Figures and Tables

**Table 1:** PICOS question with inclusion and exclusion criteria.

Concepts	Description
Population	The eligibility criteria for the search strategy are as follows: (i) Women aged ≥18 years diagnosed with breast cancer. Studies were excluded if they included: (ii) Studies in which outcome data for women with breast cancer were not reported separately from data for male patients or partners.
Intervention	Studies were included if they evaluated the effects of model- or theory-based sexual counselling on the sexual life and sexual function of women with breast cancer using sexual health-specific scales and reported mean values and standard deviations for the intervention and control groups. Partner participation in the counselling intervention was permitted, provided that sexual health outcomes were assessed and reported for women with breast cancer. Studies were excluded if they did not assess sexual life or sexual function, did not provide quantitative data, lacked a control group, or were not published in English. Studies in which sexual health outcomes for women with breast cancer could not be extracted separately were also excluded.
Comparison	Compared against standard care or placebo interventions.
Outcomes	The meta-analysis looked at measures of sexual life and sexual function in breast cancer patients.
Study design	Controlled quantitative intervention studies, including randomized controlled trials and quasi-experimental controlled studies, were eligible for inclusion.
Study selection	A total of 356 records were identified through the database searches. After removal of 202 duplicates, 154 records remained for screening. Of these, 19 full-text articles were assessed for eligibility; 9 were excluded, and 10 studies were included in the systematic review and meta-analysis.

**Table 2:** Search Concepts.

Concept 1	Concept 2	Concept 3	Concept 4
breast cancer OR mastectomy OR breast cancer survivor OR breast cancer surgery	sex OR intimacy OR sexual behavior OR sexual activity OR sexual health OR sexual wellbeing OR sexuality	(consultation OR counseling) AND (model OR modeling OR modelling OR theory)	randomized controlled trial OR RCT OR randomised controlled trial OR randomized clinical trial OR randomized controlled study

Note: The same search strategy was applied to MEDLINE, Complementary Index, Academic Search Ultimate, OAIster, and CINAHL. All search terms were restricted to the abstract field (AB). The databases were searched on 10 March 2025 and covered studies published through February 2025.

**Table 3:** Characteristics of the studies included in the systematic review and meta-analysis.

Author, Year	Country	Study Design	Model/Intervention	Participants (Sample; Age)	Measures	Sessions	Follow-Up
Abedini, 2020 [28]	Iran	Randomized controlled trial	Self-Regulation Model psychoeducational program; individual counseling	Breast cancer patients; IG = 37, CG = 36; age: IG 44.13 ± 7.72, CG 42.94 ± 6.97	ISS	3 sessions, 60–90 min	1, 2, and 3 months
Barjasteh, 2022 [31]	Iran	Randomized controlled clinical trial	Good Enough Sex (GES)-based, couple-centered group counseling vs. routine care	Breast cancer survivors; IG = 50, CG = 50; 50% of IG and 56% of CG aged 39–49 years	DASS-21; RCAC; FSFI-BC	4 weekly sessions, 90–120 min	2 and 3 months
Barjasteh, 2024 [30]	Iran	Randomized controlled clinical trial	Good Enough Sex (GES) model group counseling vs. routine care	Breast cancer survivors; IG = 50, CG = 50; 56% of IG and 50% of CG aged 39–49 years	SSSW; FSFI-6	4 weekly sessions, 90–120 min	2 and 3 months
de Almeida, 2020 [34]	Brazil	Nonrandomized pre-/post pilot controlled study	PLISSIT model counseling delivered by a nurse and a sexual therapist vs. routine care	Breast cancer survivors; IG = 10, CG = 8; age: IG 53.27 ± 5.00, CG 56.38 ± 9.43	FSFI	5 sessions	3 months
Esmkhani, 2021 [36]	Iran	Three-arm randomized controlled trial	PLISSIT model vs. Sexual Health Model group training	Breast cancer patients; PLISSIT = 25, SHMG = 25, CG = 25; age: 38.12 ± 5.56, 40.40 ± 7.29, 41.04 ± 4.40	Sexual Distress Questionnaire; EORTC QLQ-C30 V3; EORTC QLQ-BR23 V3	PLISSIT: 1–3 sessions; SHMG: 6 h workshop	6 and 12 weeks
Faghani, 2016 [32]	Iran	Quasi-experimental controlled study	PLISSIT model-based sexual counseling vs. routine care	Women with breast cancer undergoing mastectomy; IG = 43, CG = 45; mean age (combined) = 43.2	SQOL-F; FSFI	4 sessions, 90 min	4 weeks
Jalambadani, 2018 [33]	Iran	Quasi-experimental controlled study	Theory of Planned Behavior (TPB)-based educational program	Breast cancer patients; IG = 60, CG = 60; age: IG 45.75 ± 10.53, CG 46.35 ± 10.77	FSFI	Twice weekly, 60 min, for 12 weeks	12 weeks
Jamshidi, 2025 [37]	Iran	Randomized controlled trial	Sexual counseling and education based on self-efficacy theory vs. no intervention	Breast cancer patients; IG = 25, CG = 25; age: IG 42.64 ± 4.88, CG 43.44 ± 5.20	FSFI	2 group training sessions plus 3 counseling sessions	Immediately post-intervention and 1 month
Khoei, 2022 [35]	Iran	Three-arm randomized controlled trial	PLISSIT and Grouped Sexuality Education (GSE) vs. control	PLISSIT = 22, GSE = 25, CG = 18	SB	1–3 counseling sessions	6 and 12 weeks
Nazarzadeh, 2024 [29]	Iran	Randomized controlled trial	WhatsApp-based BETTER Sex model vs. routine self-care advice	IG = 45, CG = 45; age: IG 37.38 ± 7.49, CG 37.36 ± 6.51	SQOL-F; FSFI-BC	6-week counseling/assignment package	Immediately post-intervention and 1 month

Abbreviations: BETTER, BETTER Sex model; CG, control group; DASS-21, Depression Anxiety Stress Scales-21; EORTC QLQ-BR23, European Organisation for Research and Treatment of Cancer Breast Cancer Module; EORTC QLQ-C30, EORTC Core Quality of Life Questionnaire; FSFI, Female Sexual Function Index; FSFI-6, 6-item Female Sexual Function Index; FSFI-BC, Female Sexual Function Index for Breast Cancer; GES, Good Enough Sex; GSE, Grouped Sexuality Education; IG, intervention group; ISS, Index of Sexual Satisfaction; PLISSIT, Permission, Limited Information, Specific Suggestions, and Intensive Therapy; RCAC, Reproductive Concerns After Cancer; SB, Sexual Behavior; SHMG, Sexual Health Model Group; SQOL-F, Sexual Quality of Life–Female; SSSW, Sexual Satisfaction Scale for Women; TPB, Theory of Planned Behavior.

**Table 4:** GRADE assessment of the certainty of evidence for the principal outcomes.

Outcome	No. of Studies	Effect Estimate	Risk of Bias	Inconsistency	Indirectness	Imprecision	Publication Bias	Certainty of Evidence
Overall sexual life	10	Hedges’ g = 1.86 (95% CI 0.94–2.77)	Not serious	Very serious ^a^	Serious ^b^	Not serious	Not downgraded ^c^	⨁◯◯◯ Very low
Sexual function	8	Hedges’ g = 2.11 (95% CI 0.94–3.28)	Not serious	Very serious ^a^	Serious ^b^	Not serious	Not downgraded ^c^	⨁◯◯◯ Very low

^a.^ Very serious inconsistency: Certainty was downgraded by two levels because heterogeneity was extreme (I^2^ > 90%) and remained substantial in leave-one-out sensitivity analyses, indicating considerable unexplained between-study variability. ^b.^ Serious indirectness: Certainty was downgraded by one level because nine of the ten included studies were conducted in Iran, limiting the geographical and cultural applicability of the evidence to broader populations of women with breast cancer. ^c.^ Publication bias: No downgrade was applied because publication bias was not directly demonstrated. However, it could not be reliably excluded because of the small number of studies and the limited statistical power of Kendall’s tau and Egger’s regression tests. Abbreviations and symbols: CI, confidence interval; I^2^, I-squared statistic; ⨁◯◯◯, very low certainty of evidence.

## Data Availability

No new data were created or analyzed in this study. Data sharing is not applicable to this article.

## Abbreviations

PLISSIT	Permission, Limited Information, Specific Suggestions, Intensive Therapy.
SHMG	Sexual Health Model Group.
GSE	Grouped Sexuality Education.
GES	Good Enough Sex.
